# Internal 40-gauge microneedle drainage for the management of subretinal fluid in rhegmatogenous retinal detachment surgery

**DOI:** 10.1186/s40942-025-00709-x

**Published:** 2025-07-31

**Authors:** Gregor S. Reiter, Jenna Krivit, Run Zhou Ye, Raymond Iezzi

**Affiliations:** 1 Department of Ophthalmology, Mayo Clinic, 200 1st St SW, Rochester, MN 55905 USA; 2 Department of Ophthalmology, Medical University of Vienna, Vienna, Austria

**Keywords:** Microneedle drainage, Pars plana vitrectomy, Postsurgical outcomes, Rhegmatogenous retinal detachment, Subretinal fluid

## Abstract

**Purpose:**

To describe outcomes for internal microneedle drainage for the management of residual subretinal fluid (SRF) during pars plana vitrectomy (PPV) for rhegmatogenous retinal detachment (RRD).

**Methods:**

Retrospective review of patients undergoing PPV for RRD using a 40-gauge angled and beveled microneedle (Incyto, South Korea) for the aspiration and drainage of SRF during fluid/air exchange. The microneedle is inserted through the neurosensory retina into the bleb of residual SRF, removed from the subretinal space and placed onto the retina or adjacent to the subretinal entry site. Visual acuity (VA) and safety outcomes are reported.

**Results:**

Thirteen eyes from 13 patients with a median age (Q1 – Q3) of 61.12 (52.03–63.81) were included. Median visual acuity (VA) at baseline was 0.52 (0.24–0.8) logMAR (approx. 20/66) and significantly improved to 0.1 (0.02–0.26) logMAR (approx. 20/25) at the last available follow-up time point (*p* = 0.0167). Macula-off RRD had significantly worse VA at baseline 0.58 (0.3–1.0) logMAR (approx. 20/76) compared to macula-on RRD 0.15 (0.105–0.25) logMAR (approx. 20/28), *p* = 0.0196) but improved significantly to the last available follow-up (*p* = 0.0391), matching those with macula-on RRD. The use of microneedle drainage was safe and resulted in an attached retina in all 13 (100%) cases without adverse events.

**Conclusions:**

Microneedle drainage is safe, while patient outcomes are excellent. The complete aspiration of SRF might reduce complications associated with PPV for RRD and optimize visual outcomes.

**Supplementary Information:**

The online version contains supplementary material available at 10.1186/s40942-025-00709-x.

## Introduction

Rhegmatogenous retinal detachment (RRD) is a common cause for a rapid loss of vision caused by one or more peripheral breaks in the retina and the accumulation of subretinal fluid (SRF) below the neurosensory retina [1]. RRD is managed by surgical interventions that include pneumatic retinopexy, scleral buckles (SB) and pars plana vitrectomy (PPV) [2, 3]. With the development of microincision surgery, PPV alone has become increasingly performed for the repair of primary RRD. This trend may be associated with the use of cannulas that may reduce intraoperative complications associated with larger sclerotomies [4]. 

Nevertheless, postoperative complications of PPV for RRD include persistent SRF, retinal displacement with diplopia, re-detachment, endophthalmitis, formation of cataract, among others [2, 5–7]. SRF drainage is preferably performed using the original break [8, 9]. This maneuver allows for drainage if the break is not too anterior and superior. Another approach to fully drain SRF is the iatrogenic creation of a drainage retinotomy, which requires laser retinopexy for sealing [10–13]. However, recent studies have associated drainage retinotomies with increased risk for surgical failure [11, 12], or reduced visual acuity outcomes [13], whereas other studies did not show such a difference [10]. The use of heavy perfluoro-octane (PFO) can be used to effectively displace SRF from the subretinal space and drain residual SRF through the original break [8, 14]. The use of PFO however, has its own limitations and risks [15]. A recent report demonstrated the use of a 38-gauge blunt polytip cannula to drain residual SRF without the need for laser retinopexy of the drainage site to be safe [16]. A second comparison of internal drainage used a 41-gauge blunt polytip cannula to drain residual SRF, which resulted in a safe approach when compared to conventional PPV without drainage retinotomy with similar visual gains after surgery [13]. 

While there is preference to drain all SRF without the need of an additional retinotomy, a prospective study highlighted that the complete drainage of SRF might not be necessary as the retinal pigment epithelium is actively clearing residual SRF from the subretinal space [17]. However, eyes with incomplete drainage might end up with less diplopia through a more physiological aligned attachment of the macula after the surgery [2]. Complete drainage of SRF might be preferred in eyes with inferior breaks to increase the space within the eye for the tamponade and guarantee a full fill for a maximum period of time [13]. The aim of this study is to present an internal draining technique using an angled and beveled 40-gauge microneedle creating a self-sealing retinotomy to completely drain SRF from the subretinal space without the need for drainage retinotomies or endolaser-sealing retinopexies. Further, we will demonstrate that this style microneedle can leverage hydrogen bonds to drain SRF while the microneedle is applied onto the surface of the retina, adjacent to a prior subretinal entry site, potentially increasing its safety over previously reported blunt-tip 38 or 41-gauge approaches that require manual aspiration.

## Methods

This is a retrospective analysis from patients undergoing primary PPV for RRD from July 2019 to May 2024 at Mayo Clinic Rochester, MN, USA, with the use of an internal microneedle drainage technique. IRB approval was obtained, and patient informed consent was waived due to the retrospective nature of the study. The study adhered to the Declaration of Helsinki. All patients underwent surgery by the same retina specialist (RI). Patients with previous RRD surgery (e.g. primary scleral buckle) were not included in this analysis. Patients who had previously undergone failed laser retinopexy were still eligible for inclusion. Patients were assessed before surgery and during their routine follow-up scheduled at 1 week, 1 month and 3 months after surgery. Due to its retrospective and the real-world nature of this study, the patient visit closest to these time points were assessed. If available, the last available visit was also taken from the medical record.

Data is shown as median and interquartile range (Q1 – Q3), unless otherwise stated. Visual acuity was assessed at all visits with correction or pinhole and are expressed as logarithm of the minimum angle of resolution (logMAR). Counting fingers (CF) and hand motion (HM) were converted to logMAR values [18]. Differences between patients with macula-off and macula-on RRD were calculated using Wilcoxon rank sum tests. Differences between time points were investigated using the Wilcoxon signed-rank test. Groups were further split into phakic and pseudophakic eyes at baseline and into groups considering the macula involvement of the RRD. A *p*-value < 0.05 was considered significant. Due to the exploratory analysis of this cohort, no correction for multiple testing was performed.

### Surgical technique

**Fig. 1:**
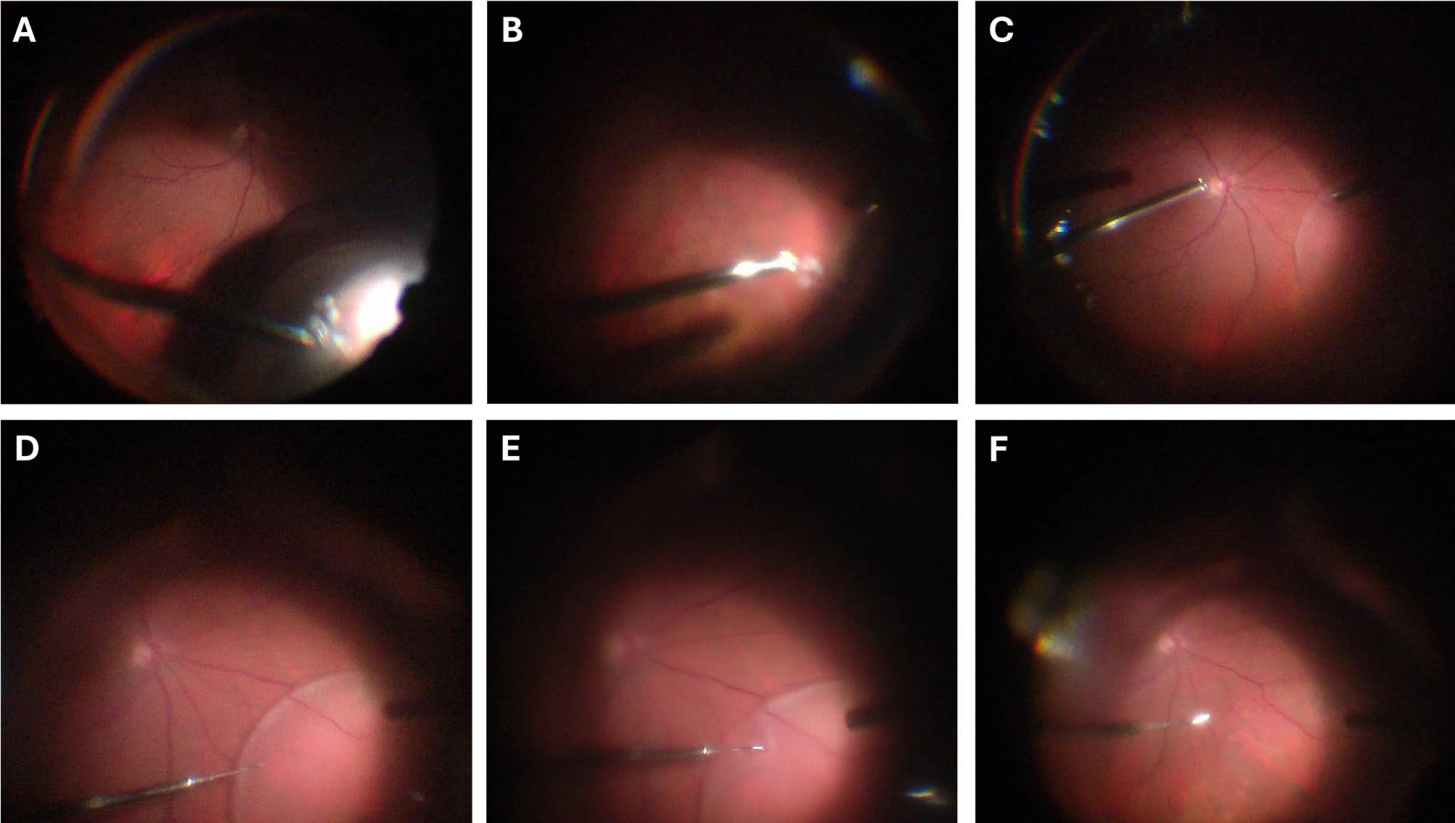
Case 1 showing the use of the angled and beveled 40-gauge microneedle in a patient with a rhegmatogenous retinal detachment in the superior nasal quadrant. (**A**) Marking of the original break with intraocular diathermy. (**B**) Internal drainage through the original break. (**C**) Persistence of subretinal fluid (SRF) can be identified. (**D**) Insertion of the 40-gauge microneedle into the subretinal space with the bevel pointing upwards. (**E**) Drainage of the retained SRF with the microneedle place onto the epiretinal surface with the bevel up (**F**) Complete attachment of the retina is achieved, and the insertion site is self-sealing

**Fig. 2:**
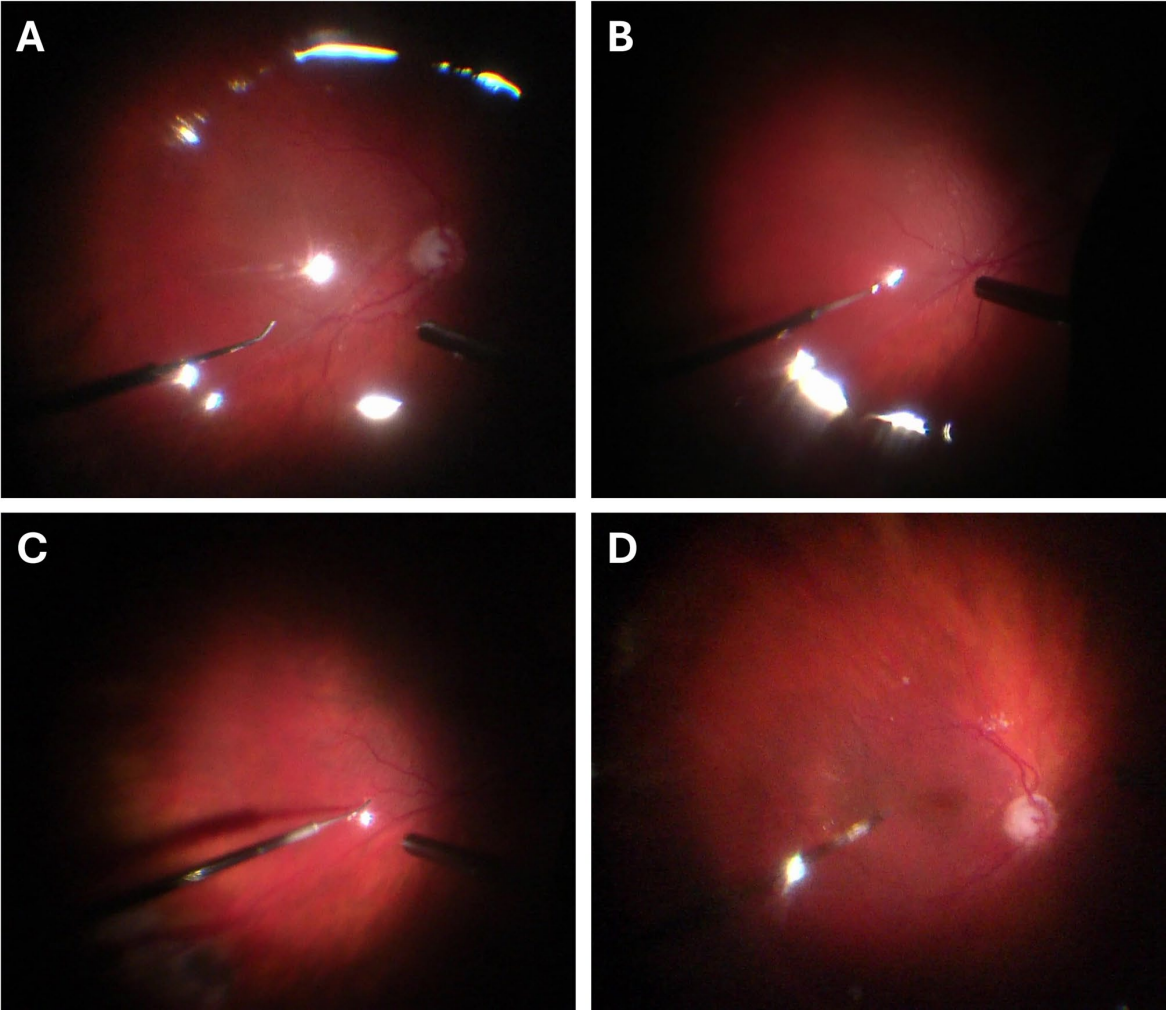
Case 2 showing the use of the 40-gauge microneedle in a patient with a rhegmatogenous retinal detachment in the inferior temporal quadrant involving the macula. (**A**) Clear view of the curvature of the needle inside the eye. (**B**) Insertion of the 40-gauge microneedle with the bevel pointing upwards. (**C**) Drainage of the retained SRF. (**D**) Attachment of the retina is achieved, and the insertion site is self-sealing. A video of the drainage is also available in the Supplemental Video 1

**Fig. 3:**
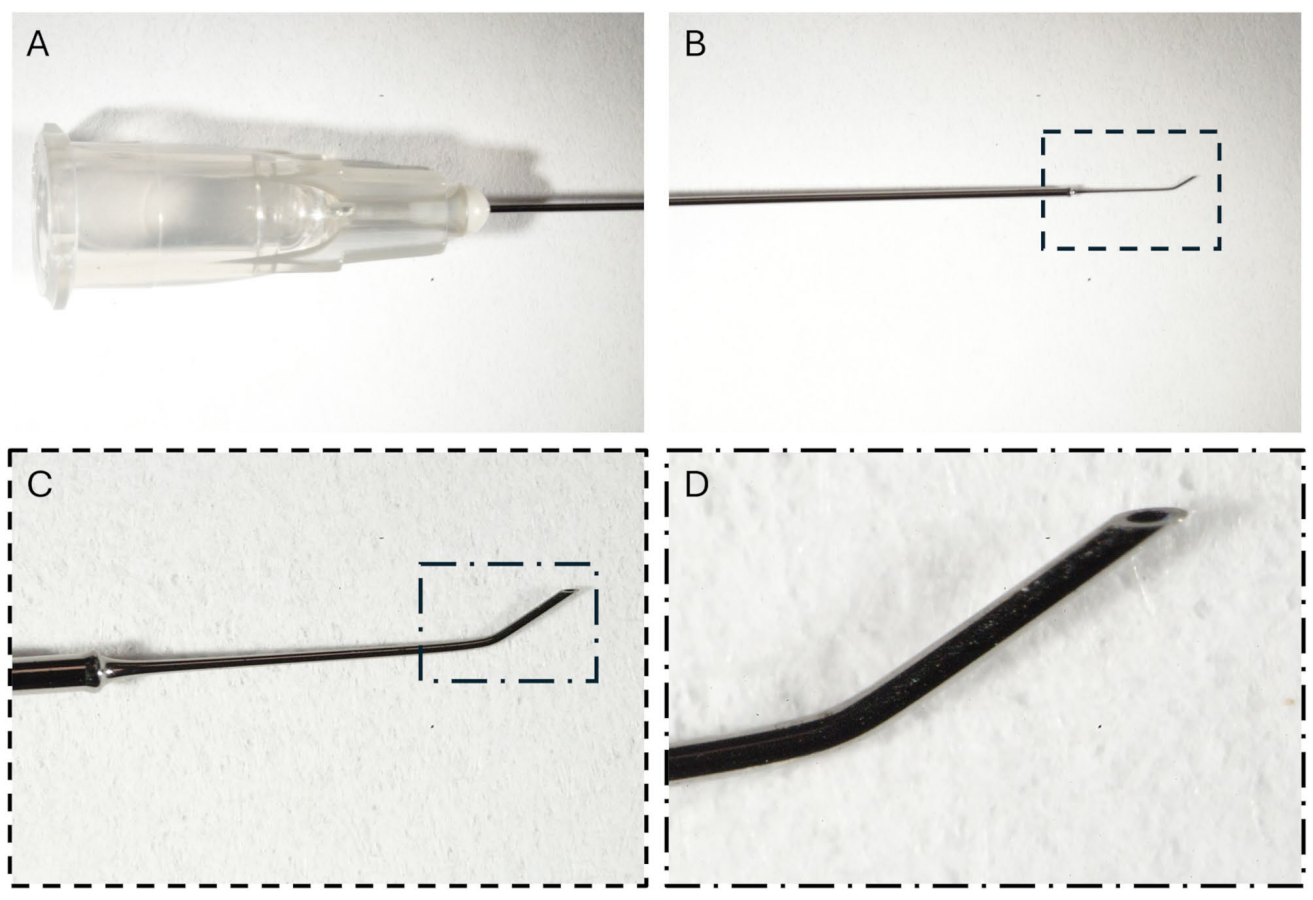
High magnification of the 40-gauge microneedle. (**A**) Hub with the 32 mm long 25-gauge shaft. (**B**) Shaft ending in the angled and beveled tip. (**C**) Magnification of the tip from **B**). (**D**) Magnification of the beveled tip with the 40-gauge opening from **C**)

All surgeries were performed by a single surgeon (RI) using widefield visualization from the RESIGHT (Carl Zeiss Meditec Inc., Dublin, CA, USA) lens system and under scleral depression by a skilled assistant. Routine RRD vitrectomy was performed by inserting three 23-gauge cannulas at the 2 and 10 o’clock positions as well as inferior temporally for the infusion, 3.5–4.0 mm back from the limbus. If attached, the posterior hyaloid was separated from the retina, if necessary, by staining the vitreous with washed and diluted triamcinolone. The retinal breaks were marked with intraocular diathermy (Fig. 1A) and fluid/air exchange was performed, draining through the original retinal breaks until the retina was reattached as much as possible (Fig. 1B). After fluid/air exchange, persistent fluid was visible under the retina (Figs. 1C and 2A), so a 40-gauge angled and beveled microneedle (Incyto, South Korea) was used to drain the remaining subretinal fluid (Figs. 1D-F, 2A-D and 3, Supplemental Video 1). Care was taken to drain in the mid-periphery or outside the arcades. The site was considered self-sealing. Endolaser retinopexy was performed 360 degrees around the original peripheral retinal breaks but not around the microneedle drainage site. In some cases, cryotherapy was used to treat the original breaks but not the microneedle drainage site. The three cannulas were removed and closed with 8 − 0 Vicryl sutures. Then the respective gas tamponade was injected into the eye with a 30-gauge needle, while a 27-gauge needle was used to vent the gas.

## Results

Thirteen eyes from 13 patients matched the inclusion/exclusion criteria and were included in this study. Demographic results of the described cohort are presented in Table 1. Median (Q1 - Q3) age at baseline (day of surgery) was 61.12 (52.03–63.81) years.

**Table 1:** Demographic description of the presented cohort of patients with rhegmatogenous retinal detachment and internal 40-gauge microneedle drainage

Demographics	
Number of eyes (Nr. of patients)	*n* = 13 (13)
Age, median (Q1 – Q3) (years)	61.12 (52.03–63.81)
Female, Nr (%)	5 (38.46)
Male, Nr (%)	8 (61.54)
Visual acuity, preoperative, median (Q1 – Q3) (logMAR)	0.52 (0.24–0.8)
**Ethnicity**	
White, Caucasian, Nr (%)	11 (84.62)
African, Nr (%)	1 (7.69)
Asian, Nr (%)	1 (7.69)
**Macula involvement of RRD**	
Macula on, Nr (%)	4 (30.77)
Macula off, Nr (%)	9 (69.23)
**Laterality**	
Left eyes, Nr (%)	9 (69.23)
Right eyes, Nr (%)	4 (30.77)
**Lens status prior to surgery**	
Phakic, Nr (%)	6 (46.15)
Pseudophakic, Nr (%)	7 (53.85)

The surgical procedures are presented in Table 2. Since the technique of internal microneedle drainage is unrelated to the combination of vitrectomy with scleral buckle and/or with phacoemulsification, all related cases that include primary vitrectomy for RRD surgery were analyzed. One patient had a previous laser retinopexy which failed during observation and was then referred for vitrectomy.

**Table 2:** Surgical procedures of the presented cohort of patients with rhegmatogenous retinal detachment and microneedle drainage

Performed surgery	
23-gauge vitrectomy, Nr (%)	9 (69.23)
23-gauge vitrectomy combined with phacoemulsification, Nr (%)	1 (7.69)
23-gauge vitrectomy combined with scleral buckle, Nr (%)	3 (23.08)
**Use of intraoperative perfluoro-octane (PFO)**	
Yes, Nr (%)	5 (38.46)
No, Nr (%)	8 (61.54)
**Use of Laser/Cryotherapy**	
Laser only, Nr (%)	12 (92.31)
Cryotherapy and laser, Nr (%)	1 (7.69)
**Tamponade**	
SF6, Nr (%)	9 (69.23)
C3F8, Nr (%)	4 (30.77)

### Visual acuity outcomes

**Fig. 4:**
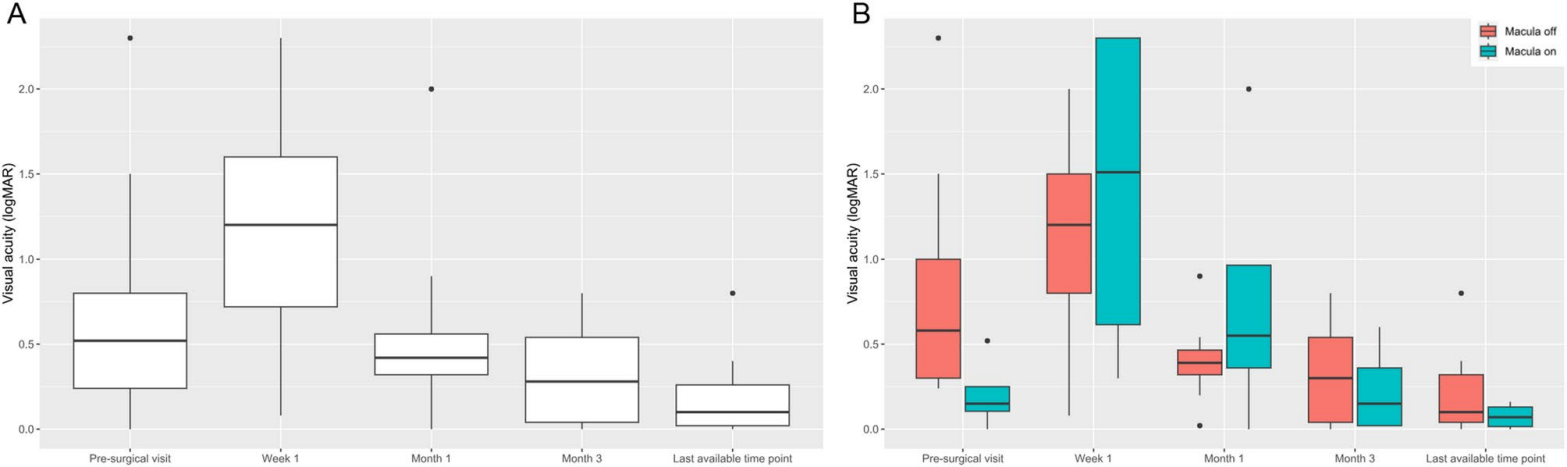
Visual acuity (logMAR) changes over time for the total cohort (**A**) and (**B**) split by the involvement of the macula into macula off rhegmatogenous retinal detachment (RRD) (red) and macula on RRD (light green)

Visual acuity results overall and split by the involvement of the macula and the overall cohort are presented in Table 3; Fig. 4. Unsurprisingly, visual acuity was significantly better in eyes without macula involvement prior to surgery (*p* = 0.0196). However, during follow-up no statistical differences were seen between the macula-off and the macula-on RRD groups (see Table 3; Fig. 3B). The median (Q1 – Q3) time of the month 3 visit as well as the last available visit was 2.8 (2.7–3.9) and 10.7 (5.0–16.8) months, respectively.

**Table 3:** Visual acuity outcomes including follow up. The median last available visit was 10.7 (5.0–16.8) months. All Phakic patients had undergone phacoemulsification with IOL implantation by the last available visit. Differences were calculated using a Wilcoxon rank sum test. Bold font represents a significant difference

Time point	Visual acuity			
	Mac off RRD (*n* = 9)	Mac on RRD (*n* = 4)	Total cohort (*n* = 13)	Difference between Mac off and Mac on RRD*
Preoperative, median (Q1 – Q3) (logMAR)	0.58 (0.3–1.0) (approx. 20/76)	0.15 (0.105–0.25) (approx. 20/28)	0.52 (0.24–0.8) (approx. 20/66)	*p* = 0.0196
Post surgery week 1, median (Q1 – Q3) (logMAR)	0.8 (0.8–1.5) (approx. 20/126)	1.51 (0.615–2.3) (approx. 20/647)	1.2 (0.72–1.6) (approx. 20/317)	*p* = 0.589
Post surgery month 1, median (Q1 – Q3) (logMAR)	0.39 (0.32–0.465) (approx. 20/49)	0.55 (0.36–0.965) (approx. 20/71)	0.42 (0.32–0.56) (approx. 20/53)	*p* = 0.461
Post surgery month 3, median (Q1 – Q3) (logMAR)	0.3 (0.04–0.54) (approx. 20/40)	0.15 (0.02–0.36) (approx. 20/28)	0.28 (0.04–0.54) (approx. 20/38)	*p* = 0.535
Post surgery last available visit, median (Q1 – Q3) (logMAR)	0.1 (0.04–0.32) (approx. 20/25)	0.07 (0.015–0.13) (approx. 20/23)	0.1 (0.02–0.26) (approx. 20/25)	*p* = 0.485

For the total cohort, no differences were found between baseline and week 1 (*p* = 0.0776) as well as baseline and month 1 (*p* = 0.5633) and baseline and month 3 (*p* = 0.0994). However, significant improvement was identified until the last available time point (*p* = 0.0167).

Similar to the total cohort, the macula-off RRD patients presented with no difference compared to baseline for week 1 (*p* = 0.441), month 1 (*p* = 0.148) and month 3 (*p* = 0.08), whereas a significant improvement was found until the last available time point (*p* = 0.0391). In comparison, the macula-on RRD patients did not differ compared to baseline at any visit (all *p* > 0.05). Due to the small number of macula-on RRD, the week 1 visit might have presented no difference as well (*p* = 0.125).

When splitting the data by the lens status prior to surgery, phakic eyes at baseline showed significant improvement until the last available time point (*p* = 0.0312, all other *p* > 0.05). One eye had combined vitrectomy with phacoemulsification in the initial procedure due to age-related cataract and the other five phakic eyes received cataract surgery after the month 3 visit. Therefore, all phakic eyes were pseudophakic at the last available time point.

### Adverse events

Primary retinal reattachment was achieved for all 13 eyes (100%). No adverse events were noticed during follow-up with relevance to the internal microneedle drainage. One eye received combined phaco-vitrectomy during the primary surgery. The five remaining phakic eyes developed cataract related due to the vitrectomy procedure with gas tamponade and were operated on between the month 3 visit and the last available time point. No signs of additional inflammation or hemorrhage were reported.

## Discussion

Success in RRD surgeries depends on the closure of the original break. Residual SRF in inferior breaks should be avoided to increase the space for the tamponade or in patients unable to maintain face down positioning [13]. However, the creation of an iatrogenic drainage retinotomy might limit the success of vitrectomy for RRD [12]. Herein, we present a technique to drain most residual SRF from beneath the neurosensory retina with an angled and beveled 40-gauge microneedle. In contrast to the previously reported 38-gauge and 41-gauge polytip subretinal needles that are blunt at the tip [13, 19], this 40-gauge needle is slightly angled with a beveled tip pointing upwards (Fig. 3). The tip of this needle can therefore be placed on top of the drainage site and the residual SRF can be drained while gently pushing down on the retina with the outside curvature while fluid/air exchange is performed (Fig. 2C, Supplemental Video 1). This avoids the risk of movement while keeping a blunt tip inside the subretinal space, which increases the chances to enlarging the drainage site’s size. This might result in a retinotomy that is no longer self-sealing and increases the risk of the failure of the surgery. The sharp beveled tip might provide a better-sealing drainage site, although this has not been proven. Although beveled, with its angled tip the risk of puncturing the retinal pigment epithelium and the choroid at the end of drainage is considerably reduced by using the needle to drain subretinal fluid from the epiretinal surface, making the technique with the 40-gauge angled microneedle safe (Supplemental Video 1). In addition, extraction is achieved by leveraging the hydrogen bonds of the SRF itself.

Unlike conventional retinotomies, which require cryo- or laser retinopexy to seal the iatrogenic break, microneedle drainage potentially allows for drainage closer to the posterior pole and the removal of most SRF, especially in cases involving the macula. More posterior retinotomies with additional laser retinopexies may affect the patient’s postoperative visual field. Our successful prevention of redetachment demonstrates the safety of this procedure.A potential difficulty with internal microneedle drainage may occur with increasing viscosity of the SRF, which may be associated with an increase in the duration of RRD. Studies have shown that extended time of RRD is also associated with prolonged time of SRF clearance [20, 21]. The presented microneedle has a lumen of 40 gauge (approx. 0.1 mm), therefore more viscous SRF may be more difficult to drain. This was experienced in one of these cases and although complete drainage was achieved, it may have taken longer to achieve full reattachment. To overcome this problem, it is recommended that all SRF from the original break is drained first. If there is persistence of SRF, it is likely to be from the infusion itself and will be less viscous and therefore easier to drain with the microneedle. This might occur even more frequently with more anterior breaks. However, previous reports were able to efficiently drain SRF with a 41-gauge cannula [13]. 

The complete drainage of SRF has several advantages. First, complete drainage might reduce the time of rehabilitation for patients while increasing the likelihood of adequate chorioretinal apposition at the site of break. Secondly, this technique may decrease the need for strict facedown positioning which can increase patient satisfaction after surgery. Thirdly, larger amounts of residual SRF might disturb the endolaser’s ability to create long-lasting retinopexy scars, a possible reason for the failure of the surgery when the edges of the break were not sealed until chorioretinal adhesion develops. Lastly, the increased volume gained within the eye by the total absorption of SRF creates more space for endo-tamponades, which is especially important in retinal breaks that occur in the inferior quadrants. Furthermore, the efficacy of scleral buckling must be acknowledged as a potential treatment in suitable cases, especially in inferior breaks. However, scleral buckling is being used less frequently in contemporary vitreoretinal surgery, particularly among surgeons trained primarily in PPV techniques. We believe our technique can serve as a useful adjunct to, not a replacement for, sound surgical fundamentals.

In contrast, the limitation of the internal microneedle drainage technique is its increased surgical time and the use of an additional instrument, increasing the overall cost of an already expensive surgery. In our experience, this 40-gauge needle has become a new option in the toolbox for draining residual SRF. It is preferable to drain the SRF through the original break when such an option is accessible. If only minimal residual SRF is present, we believe that this 40-gauge needle approach is not necessary, as we do not expect much benefit over leaving minimal amounts of residual fluid. However, as described above, in cases where substantial SRF cannot be drained through the original break, this approach has become an important method to release fluid from under the retina. We reserve this approach for these cases.

A limitation of our study is its small cohort and retrospective nature, including not fully standardized visual acuity assessments and deviations from scheduled routine visits, but these did not affect the conclusions of this work. Another limitation is the lack of a randomized control group.

## Conclusions

In conclusion, we report outcomes for draining residual SRF using an angled and beveled 40-gauge microneedle which, when used as described, is self-sealing and therefore unlike conventional drainage retinotomies. Although our cohort was small, optimal visual gains were achieved in these patients with visual acuity results of macula off RRD comparable to macula on RRD and excellent functional outcomes compared to the literature. Prospective studies are therefore encouraged to further investigate the use of internal microneedle drainage.

## Electronic supplementary material

Below is the link to the electronic supplementary material.

**Supplementary Material 1**: **Supplemental Video 1**: Drainage of subretinal fluid using the internal 40-gauge microneedle.

## Data Availability

The data supporting the findings of this study are available from the corresponding author upon reasonable request.

## Abbreviations

CF: Counting fingers
HM: Hand motion
LogMAR: Logarithm of the minimum angle of resolution
PFO: Perfluoro-octane
PPV: Pars plana vitrectomy
RRD: Rhegmatogenous retinal detachment
SB: Scleral buckle
SRF: Subretinal fluid

## References

[1] Mitry D, Charteris DG, Fleck BW, Campbell H, Singh J. The epidemiology of rhegmatogenous retinal detachment: geographical variation and clinical associations. Br J Ophthalmol. 2010;94(6):678–84. 19515646 10.1136/bjo.2009.157727

[2] Bansal A, Naidu SC, Marafon SB, Kohler JM, In S, Mahendrakar PA, Garima, Kashyap H, Susavar P, Bhende M, et al. Retinal displacement after scleral buckle versus combined buckle and vitrectomy for rhegmatogenous retinal detachment: ALIGN scleral buckle versus Pars plana vitrectomy with scleral buckle. Ophthalmol Retina. 2023;7(9):788–93. 37217137 10.1016/j.oret.2023.05.012

[3] Grad JR, Hatamnejad A, Huan PW, Popovic MM, McKay BR, Kertes PJ, Muni RH. SURGICAL DRAINAGE METHODS DURING PARS PLANA VITRECTOMY FOR RHEGMATOGENOUS RETINAL DETACHMENT: A systematic review and Meta-Analysis. Retina. 2024;44(5):747–55. 38437843 10.1097/IAE.0000000000004083

[4] Chen GH, Tzekov R, Jiang FZ, Mao SH, Tong YH, Li WS. Iatrogenic retinal breaks and postoperative retinal detachments in microincision vitrectomy surgery compared with conventional 20-gauge vitrectomy: a meta-analysis. Eye (Lond). 2019;33(5):785–95. 30560911 10.1038/s41433-018-0319-5 PMC6707291

[5] Azaizy M, Khalil HEM, Leila M, Akl NS, Mohammed SI. Evaluation of post-operative foveal location and microstructural changes after Pars plana vitrectomy for rhegmatogenous retinal detachment using enhanced-depth imaging optical coherence tomography. Int J Retina Vitreous. 2024;10(1):88. 39574210 10.1186/s40942-024-00609-6 PMC11580562

[6] Kasetty VM, Monsalve PF, Sethi D, Yousif C, Hessburg T, Kumar N, Hamad AE, Desai UR. Cataract progression after primary Pars plana vitrectomy for uncomplicated rhegmatogenous retinal detachments in young adults. Int J Retina Vitreous. 2024;10(1):19. 38383511 10.1186/s40942-024-00538-4 PMC10882894

[7] Hosseini S, Daraee G, Shoeibi N, Bakhtiari E, Ansari-Astaneh MR, Abrishami M, Motamed Shariati M. Incidence rate and clinical characteristics of acute endophthalmitis following 23-gauge Pars plana vitrectomy. Int J Retina Vitreous. 2022;8(1):85. 36544227 10.1186/s40942-022-00435-8 PMC9768931

[8] Hahn P, Eliott D. ASRS GLOBAL TRENDS in Retina. In. 2021.

[9] Yamaguchi M, Ataka S, Shiraki K. Subretinal fluid drainage via original retinal breaks for rhegmatogenous retinal detachment. Can J Ophthalmol. 2014;49(3):256–60. 24862771 10.1016/j.jcjo.2014.03.001

[10] Fukuyama H, Ishikawa H, Gomi F, Japan-Retinal Detachment Registry G. Impact of drainage retinotomy on surgical outcomes of retinal detachment: insights from the Japan-Retinal detachment registry. Sci Rep. 2024;14(1):7795. 38565682 10.1038/s41598-024-58453-5 PMC10987606

[11] McDonald HR, Lewis H, Aaberg TM, Abrams GW. Complications of endodrainage retinotomies created during vitreous surgery for complicated retinal detachment. Ophthalmology. 1989;96(3):358–63. 2469050 10.1016/s0161-6420(89)32902-2

[12] Ohara H, Yuasa Y, Harada Y, Hiyama T, Sadahide A, Minamoto A, Hirooka K, Kiuchi Y. Drainage retinotomy is a risk factor for surgical failure after Pars plana vitrectomy in patients with primary uncomplicated rhegmatogenous retinal detachment. Retina. 2022;42(12):2307–14. 36394886 10.1097/IAE.0000000000003608

[13] Desira M, Ruiz T, Comet A, Matonti F, Conrath J, Gravier-Dumonceau R, Delaporte C, Morel C, Devin F, David T, et al. Transretinal puncture with a 41G cannula for posterior residual subretinal fluid in fovea-off retinal detachments treated by vitrectomy vs fluid tolerance vs other conventional drainage techniques: A comparative study. Retina. 2025;45(2):257–68. 39454073 10.1097/IAE.0000000000004309

[14] Wurtz M, Dormegny L, Muller C, Bourcier T, Ballonzoli L, Gaucher D, Saleh M. Perfluorocarbon liquid use during vitrectomy for Macula-Off retinal detachment has no impact on macular folds and metamorphopsia. Retina. 2024;44(11):1891–8. 39102743 10.1097/IAE.0000000000004220

[15] Tewari A, Eliott D, Singh CN, Garcia-Valenzuela E, Ito Y, Abrams GW. Changes in retinal sensitivity from retained subretinal perfluorocarbon liquid. Retina. 2009;29(2):248–50. 18854788 10.1097/IAE.0b013e318188c7ea

[16] Belin PJ, Mundae R, Tzu JH, Chang E, Parke DW. 3rd: external drainage of subretinal fluid during rhegmatogenous retinal detachment repair. Retina. 2021;41(9):1828–32. 33512898 10.1097/IAE.0000000000003136

[17] Chen X, Zhang Y, Yan Y, Hong L, Zhu L, Deng J, Din Q, Huang Z, Zhou H, Complete subretinal fluid drainage is not necessary during vitrectomy surgery for macula-off rhegmatogenous retinal detachment with peripheral breaks. A prospective, nonrandomized comparative interventional study. Retina. 2017;37(3):487–93. 27429377 10.1097/IAE.0000000000001180

[18] Lange C, Feltgen N, Junker B, Schulze-Bonsel K, Bach M. Resolving the clinical acuity categories hand motion and counting fingers using the Freiburg visual acuity test (FrACT). Graefes Arch Clin Exp Ophthalmol. 2009;247(1):137–42. 18766368 10.1007/s00417-008-0926-0

[19] Bansal A, Naidu SC, Figueiredo N, Alrabiah M, Hamli H, Wong DTW, Muni RH, Altomare F. 38-Gauge Cannula-Based endodrainage of posteriorly trapped intraoperative subretinal fluid during vitrectomy for retinal detachment. Ophthalmol Retina. 2024;8(7):727–9. 38527570 10.1016/j.oret.2024.03.018

[20] Do JR, Park DH, Shin JP, Kang YK. Effect of external subretinal fluid drainage on persistent subretinal fluid after scleral buckle surgery in macula-involving rhegmatogenous retinal detachment. Sci Rep. 2023;13(1):22176. 38093092 10.1038/s41598-023-49719-5 PMC10719269

[21] Mansour AM, Lopez-Guajardo L, Belotto S, Lima LH, Charbaji AR, Schwartz SG, Wu L, Smiddy WE, Ascaso J, Jurgens I, et al. Recovery course of persistent posterior subretinal fluid after successful repair of rhegmatogenous retinal detachment. Eur J Ophthalmol. 2024;34(4):1217–27. 37901895 10.1177/11206721231210693

